# The Role of Epstein-Barr Virus in the Pathogenesis of Autoimmune Diseases

**DOI:** 10.3390/medicina61071148

**Published:** 2025-06-25

**Authors:** Natalia Morawiec, Bożena Adamczyk, Aleksandra Spyra, Mikołaj Herba, Sylwia Boczek, Natalia Korbel, Piotr Polechoński, Monika Adamczyk-Sowa

**Affiliations:** Department of Neurology, Faculty of Medical Sciences in Zabrze, Medical University of Silesia in Katowice, 3 Maja 13-15, 41-800 Zabrze, Poland; bozena.m.adamczyk@gmail.com (B.A.); aleksandra1828@gmail.com (A.S.); mikoaj.herba9@gmail.com (M.H.); sylwiaboczek10@gmail.com (S.B.); s84033@365.sum.edu.pl (N.K.); s83084@365.sum.edu.pl (P.P.); m.adamczyk.sowa@gmail.com (M.A.-S.)

**Keywords:** autoimmune diseases, autoimmunity, Epstein-Barr virus, molecular mimicry

## Abstract

*Background and Objectives*: The Epstein-Barr virus (EBV) belongs to the gamma herpesviruses family. Evidence from the literature suggests that EBV initiates immune responses and the production of antibodies. Chronic or recurrent EBV infections may be associated with autoimmune diseases such as systemic lupus erythematosus, Sjögren’s syndrome, rheumatoid arthritis, multiple sclerosis, or inflammatory bowel diseases. This review aims to establish the role of EBV in the development and progression of autoimmune diseases. *Materials and Methods*: A literature search was conducted using PubMed, PMC, Google Scholar, and SCOPUS. Relevant studies, including meta-analyses, case-control studies, literature reviews, cross-sectional studies, and longitudinal studies, were identified through titles and abstracts screening for a comprehensive analysis. *Results*: Our study revealed a strong association between EBV infection and several autoimmune diseases, including multiple sclerosis, systemic lupus erythematosus, rheumatoid arthritis, and inflammatory bowel disease. Epstein-Barr virus seropositivity was significantly higher in affected individuals. Elevated EBV-specific antibodies correlated with disease onset and severity. EBV DNA and latency proteins were detected in diseased tissues, alongside immune dysregulation and molecular mimicry mechanisms. *Conclusions*: Our findings highlight that EBV may be an important factor in autoimmune disease pathogenesis, contributing to immune activation and tissue damage. Further research is needed to explore EBV-targeted therapies and their potential in preventing or managing autoimmune diseases.

## 1. Introduction

The Epstein-Barr virus (EBV) belongs to the gammaherpesviruses family. The infection is prevalent worldwide. Most exposures to EBV occur during childhood. In developing countries, most children are seropositive by the age of five. In highly developed countries the onset of infection is delayed, but almost all adults are seropositive. The virus is transmitted through contact with secretions, including saliva, semen, and blood [1,2]. EBV primarily targets B cells. Other cells can be affected in individuals with immunological disorders [1]. EBV is a direct cause of infectious mononucleosis (IM). The infection is typically observed in young adults, between the ages of 15 and 24 and is mostly subclinical. It may present with fever, pharyngitis, hepatitis, and lymphadenopathy. In transplant patients, EBV infection can manifest as post-transplant lymphoproliferative disorder (PTLD) [3].

Increasing evidence indicates that EBV may play a role in the pathogenesis of autoimmune diseases (ADs) [2,4,5]. Chronic or recurrent EBV infection may be associated with systemic lupus erythematosus (SLE) and Sjögren’s syndrome (SjS), where it primarily affects epithelial cells. The infection of B cells has been implicated in the pathogenesis of rheumatoid arthritis (RA) and multiple sclerosis (MS) [2,4]. Numerous case reports and studies highlighted a high prevalence of EBV infection among patients with inflammatory bowel diseases (IBDs) [5]. The ability of EBV to migrate between epithelial cells and B cells contributes to the occurrence of overlapping syndromes [2].

The aim of this review was to establish the role of EBV infection in the development and progression of ADs. By analyzing current research, this study seeks to provide a comprehensive understanding of the mechanisms by which EBV contributes to ADs.

## 2. Materials and Methods

A comprehensive literature search was conducted using PubMed, PMC, Google Scholar, and SCOPUS databases. In this study, a temporal criterion was established, limiting the inclusion of scientific publications to those from the past five years. However, selected seminal works from 2013 and 2015 were also incorporated to enhance the comprehensiveness of the analysis. Search terms included “EBV epidemiology”, “prophylaxis of EBV”, “EBV multiple sclerosis”, “EBV Crohn’s disease”, “EBV population”, “EBV autoimmune disease”, “EBV IgA nephropathy”, “EBV myasthenia gravis”, “EBV Graves’ disease”, “EBV Hashimoto thyroiditis”, “EBV systemic autoimmune disease”, “EBV diagnostics”, and “EBV immunology”. Titles and abstracts were initially screened to identify relevant studies. Full texts of potentially eligible articles were reviewed for final inclusion. To obtain a wider understanding of the subject and comprise a significant amount of research data, we included a variety of types of studies—meta-analyses, case-control studies, literature reviews, cross-sectional studies, and prospective, longitudinal studies. Studies were included if they focused on the epidemiology, pathogenesis, clinical manifestations, and diagnostics of EBV and possible connections between EBV and ADs.

## 3. Epidemiology

The EBV is transmitted mainly through the oral route. It has been observed that blood transfusions and organ transplants can also facilitate the spread of EBV [6]. Approximately 95% of healthy adults are seropositive for EBV [7]. A seroprevalence study conducted on the United Kingdom population revealed that EBV seroprevalence increases with age. In children aged 1 to 4.9 years, seropositivity rates were reported at 67.8% for girls and 72.0% for boys. This seroprevalence is significantly higher in the 20–25-year age group, with 96.4% of females and 95.5% of males testing seropositive. Furthermore, it has been established that each additional year of age is associated with a 12% increase in the odds of being seropositive [8].

In contrast to the patterns observed in Europe and North America, individuals in Asia tend to acquire EBV at an earlier age. In this region, seroprevalence increases rapidly with age, exceeding 80% by the age of 5 and reaching 90% by the ages of 7 to 8. Studies conducted in North America and Europe indicate a more gradual increase in seroprevalence, not reaching 90% until the age of 22 [9].

In developing countries, primary infection commonly occurs during early childhood, with widespread seroconversion typically observed by the age of 3–4 years. It has been demonstrated that children from various geographic regions, including Thailand, Turkey, and Ghana, are more likely to be seropositive for EBV when living in low-income, overcrowded households [10]. According to Lang et al. early EBV infection patterns were consistent across three genetically distinct Melanesian populations, despite differences in living conditions and social practices. In all three groups, mothers commonly chewed food before feeding it to their children, suggesting that both direct and indirect exposure to saliva may facilitate early EBV transmission. Indirect transmission pathways may include contact with contaminated objects, such as unclean toys [11]. These findings indicate that socioeconomic factors affect EBV seroprevalence. Developmental differences between countries influence the age of primary infection.

According to Dunmire et al. primary EBV infection results in typical IM in 75% of young adults (18–22 years old) [12]. Approximately 15% of individuals exhibit atypical symptoms, while 10% remain completely asymptomatic [13]. Due to the logistical challenges associated with conducting prospective studies in young children, the incidence and impact of primary EBV infection in preadolescents have not been extensively investigated. It is generally believed that most primary infections in children before puberty are asymptomatic [12].

Over 1% of malignancies, including Hodgkin’s lymphoma, gastric carcinoma, nasopharyngeal carcinoma, and PTLDs in people worldwide, are associated with EBV [6,14]. This association highlights the virus’s significance in public health. Lanz et al. revealed that 20–25% of MS patients have antibodies against GlialCAM and EBV nuclear antigen 1 (EBNA1) [15]. Zhou et al. reported that 8.4% of IBD patients exhibited detectable levels of EBV DNA in their blood [16].

The development of a vaccine against EBV remains the most promising approach for controlling the epidemic. However, despite several decades of research, no vaccine has yet been approved [17].

## 4. Diagnostics of EBV Infection

Several diagnostic methods are currently available for the identification of EBV infection. These include serological tests [heterophile antibody test, EBV-specific antibody tests, immunoglobulin (Ig) G avidity test], molecular methods [polymerase chain reaction (PCR) and other nucleic acid amplification methods], in situ hybridization (ISH), and viral cell culture. Acute primary EBV infection is usually diagnosed based on history, physical examination, and the presence of viral capsid antigen (VCA) IgG and IgM antibodies and EBNA1 IgG. In the event of indirect results, PCR, Western blot, heterophile antibody (HAb) test, and avidity test are used [18].

### 4.1. Serological Tests

Viral proteins such as EBNA1, EBNA2, VCA proteins p23 and p18, early antigen diffuse (EAD), glycoprotein (gp) 350, and BamHI-A rightward frame 1 (BARF1) are used to detect EBV antibodies [2].

#### 4.1.1. Heterophile Antibody Test

The heterophile antibody test (HAb) is recognized as a prevalent serological assay for identifying both primary and recurrent EBV infections. Current estimates indicate that 85–90% of adults and adolescents will test positive during an EBV infection, with approximately 50% of cases displaying positivity within the first week. This test can differentiate between late primary infection and reactivation. However, it is not specific and may provide false positive results for non-EBV infections (hepatitis, rubella, malaria, and HIV), malignancies, and autoimmune diseases [2,18].

#### 4.1.2. EBV-Specific Antibody Tests

Tests for specific anti-EBV antibodies include VCA IgG, EBNA1 IgG, IgM, and EAD IgG. There are three methods for routine EBV diagnosis: immunofluorescence assays (IFAs), enzyme immunoassays (EIAs), and Western blot assays. The gold standard for the diagnosis of primary EBV infection is still IFA. EIA is considered a viable alternative in terms of sensitivity and specificity [18]. EBV infection can be confirmed by blood testing for antibodies and by assessing VCA IgG, VCA IgM, and EBNA1 IgG. This allows us to distinguish acute from past infection. The presence of VCA IgM and VCA IgG in the absence of EBNA1 IgG indicates an acute primary infection, whereas the presence of VCA IgG and EBNA1 IgG without VCA IgM usually indicates a past infection (Table 1). VCA IgM typically appears prior to VCA IgG and dissipates within a few weeks. However, it may persist for several months. VCA IgG usually appears at the onset of acute infection and persists throughout life [19]. EBNA2 IgG antibodies appear early in the course of infection, whereas EBNA1 IgG antibodies can usually be detected 3–4 weeks after the onset of symptoms, indicating past infection [18]. IgA levels can be used to determine the burden of epithelial infection [2]. The detection of EBV reactivation can be accomplished through serological methods—during the current or recent reactivation, VCA IgG levels increase, and EAD IgG is detected [20]. In addition to IgG and IgM, which are commonly used in the serological assessment of EBV infection, IgA antibodies—particularly against EBNA1—may also hold diagnostic and prognostic value, as demonstrated in nasopharyngeal carcinoma and potentially other EBV-associated conditions [21].

#### 4.1.3. Avidity Test

The avidity evaluates the binding strength of antibodies to multivalent antigens. The analysis of the avidity of individual EBV IgG antibodies enables a more precise estimation of the dates of infection. The avidity gradually increases throughout the course of infection. IgG VCA avidity testing allows us to differentiate the primary infection from past infection in EBNA1 IgG or VCA IgM negative cases [18].

### 4.2. Molecular Methods

Methods detecting the presence of EBV genetic material in infected individuals include direct sequencing, ISH, and quantitative real-time PCR (qPCR) [2,18]. PCR is used for early diagnosis of EBV-related diseases. Due to its high sensitivity, qPCR is particularly useful in the diagnosis of acute infection and asymptomatic reactivation, as well as in monitoring patients with a high risk of developing EBV-related diseases or immunocompromised individuals [18]. The presence of EBV DNA in blood signifies active viral replication, thereby indicating EBV infection or reactivation [22]. EBV DNA detection is especially useful in the early stages of acute infection due to its higher sensitivity than serological tests or IgG avidity. EBV DNA testing is highly useful in immunocompromised patients, in patients with negative or inconclusive serological test results, and in patients with EBV-related malignancies. EBV DNA can be detected in latently infected EBV cells [18]. A prior infection or reactivation can also be demonstrated through PCR analysis of saliva [2].

### 4.3. In Situ Hybridization

In situ hybridization (ISH) is the gold standard for the diagnosis of EBV-associated carcinoma [18]. EBV-encoded RNA hybridization and EBV latent membrane protein 1 (LMP1) immunostaining are commonly utilized for the detection of latent EBV in tissues affected by PTLD or in enlarged nodes in patients with IM [2].

## 5. Immunology

### 5.1. The Structure of the EBV Virus

The EBV has an outer lipid envelope that contains both host cell membrane proteins and viral proteins (viral tegument, capsid, and genome). The main proteins of the viral envelope are glycoproteins (gps). This envelope facilitates viral replication and evasion of the host immune response [2]. EBV virus adapted well to humans, the only known host [23]. In vitro, it shows expression of nine viral proteins associated with latency: six nuclear proteins (EBNA1, EBNA2, EBNA3A, EBNA3B, EBNA3C, and EBNA-LP), three membrane proteins (LMP1, LMP2A, and LMP2B), and two small RNA molecules—Epstein-Barr encoding regions (EBER-1 and EBER-2) [24]. The virus primarily targets nasopharyngeal and oral pharyngeal epithelial cells and B lymphocytes [25]. Due to the expression of the main entry receptor-complement component receptor-2 (CR2, CD21)_B lymphocytes are the main target of EBV [26]. EBV initially infects oral epithelial cells, then penetrates the oral mucosa, reaching the lymphoid tissue of the tonsils, where it infects host B lymphocytes [27]. The life cycle of EBV includes three phases: primary infection, latency, and lytic reactivation [2]. The latent phase allows the virus to survive after the acute infection, leading to a lifelong infection in the host [28].

### 5.2. The Infection of Epithelial Cells and B Lymphocytes

EBV infection of epithelial cells occurs through several key proteins: EAD (BMFR1), glycoprotein B (gB), glycoprotein H (gH), and glycoprotein L (gL). The virus attaches to the cell surface mainly through the interaction of gH/gL with the ephrin A2 receptor (EphA2), gH/gL and gB with integrins αvβ5/αvβ6/αvβ8, and through BMRF1, which interacts with integrins β1. The EBV gP350/220 binds to CR2 and CR1 (CD35) receptors. This protein plays a significant role in the attachment of the virus to the epithelial cells. After binding to integrins or EphA2, gH/gL undergoes a shape change that enables it to interact with trimeric gB, which then helps the virus enter the cell [2,22]. EBV uses envelope proteins such as gP350/220, gB, gH, gL, and gP42 to infect B lymphocytes. The mechanism of infection of B lymphocytes is mediated by gP350/220. gP350/220 binds to CR2 and CR1 together with gP42. That results in the formation of a complex with the major histocompatibility complex II (MHC-II). The gH/gL complex forms a fusion complex with gP42-MHC-II, resulting in a conformational change in gH/gL. Consequently, the trimeric gB alters its conformation and facilitates the fusion of the viral membrane with the endosome membrane, thereby enabling EBV to enter the cytoplasm of the host cell [2]. The internalized virions then move to the cell nucleus, where the EBV genome is deposited. Successful infection is accomplished through the sequential expression of six EBV EBNAs and two LMPs. Then EBV can enter a lytic cycle or a latency [27]. During latency, viral proteins such as EBNA-2 and LMP-1 rewire B-cell signaling, metabolism, and transcription by activating MYC, NF-κB, and PI3K pathways. That leads to abnormal proliferation and differentiation [29]. This dysregulation promotes activation-induced cytidine deaminase expression, aberrant class-switch recombination, and loss of tolerance. Additionally, molecular mimicry between EBV proteins (e.g., EBNA-1, EBNA-2) and nuclear self-antigens facilitates epitope spreading, further enhancing autoreactive B-cell and T-cell responses—ultimately contributing to autoantibody production.

### 5.3. Lytic Phase

After entering a cell, the viral genetic material is transcribed, translated, and replicated to synthesize new viruses [2]. The lytic phase of the virus is controlled by the genes BZLF-1 and BRLF-1, which encode the transactivating proteins Zta and Rta. This cycle consists of three phases: early-immediate, early, and late. It usually occurs in epithelial cells, where virions are produced and where the host cell undergoes lysis [25]. The VCA p18 and VCA p23 antigens induce encapsulation of the EBV genome in infected cells, which protects EBV DNA and RNA. The early restricted antigen (EAR) prevents the premature death of host cells during the process of virus production [19]. Newly produced virions, capable of infecting other cells, exit the cell via exocytosis [2,19].

### 5.4. The Body’s Response to EBV Infection

EBV infection results in the activation of intracellular and extracellular antiviral mechanisms. The virus uses complex mechanisms to avoid detection and elimination by the immune system. That includes a host cell-derived envelope, which has numerous membrane proteins responsible for evading immune responses and for entering the latency phase. EBV latency is characterized by minimal expression of viral genes and presentation of viral peptides to the immune system [2,26]. Development of latency requires evading the immune response, which EBV achieves by blocking interferon (IFN) gene expression, inhibiting complement activation, and inactivating the cytotoxic function of CD8+ lymphocytes [26]. In deep latency, only EBNA1 is expressed, ensuring the maintenance of the EBV genome and its replication with the chromosomes of host cells. To avoid the presentation of EBNA-derived peptides on MHC-I, EBNA1 contains a characteristic AG repeat sequence that interferes with proteasome processing and interacts with nucleolin to inhibit its expression. EBNA1 also contains characteristic RG repeat sequences that may play a role in immune evasion. Latency mainly affects B cells but can also occur in epithelial cells [2]. Different latent forms of EBV have been identified, differing in their transcription profile of non-coding RNAs and protein-coding mRNAs. Different latent forms of EBV have been identified, differing in their transcription profiles of non-coding RNAs and protein-coding mRNAs. Type 0 latency is observed in healthy individuals, where EBV persists in non-dividing memory B cells with highly restricted gene expression limited to two non-coding RNAs (EBER1 and EBER2) and viral miRNAs. Upon reactivation or under certain stimuli, these cells can switch to latency type I, which is required for episome replication. Latency I is specifically associated with EBV-positive Burkitt’s lymphoma, where EBNA1 is the only expressed protein, enabling viral genome maintenance. In this form, latent EBV genomes can proliferate in memory B cells. Gly-Ala repeats in EBNA1 inhibit antigen processing, preventing CD8+ T-cell recognition of infected cells. Latency type II, characterized by expression of LMP1 and LMP2A, is observed in nasopharyngeal carcinoma and Hodgkin’s lymphoma. These latent membrane proteins can drive B-cell activation and proliferation. In latency type III, all latent gene products are expressed—including six EBNAs, three LMPs, two EBERs, and miRNAs—and this pattern is typically found in lymphoblastoid cell lines, acute infectious mononucleosis, and EBV-associated lymphoproliferative disorders in immunocompromised individuals. This form of latency may mediate the activation and transformation of naive B cells [20,27].

### 5.5. Reactivation

In altered cellular immunity, such as stress, infection, and immunosuppression, EBV may be reactivated, resulting in the production of infectious virions [22]. Lytic reactivation from plasma cells leads to the generation of amplified EBV and the infection of additional host cells. This may contribute to the development of disease in certain individuals [2,26,27]. Possible causes of increased EBV reactivation include ineffective regulation of the latent phase or an increased transition from the latent phase to the lytic phase [20].

### 5.6. Autoimmunity

**Figure 1:**
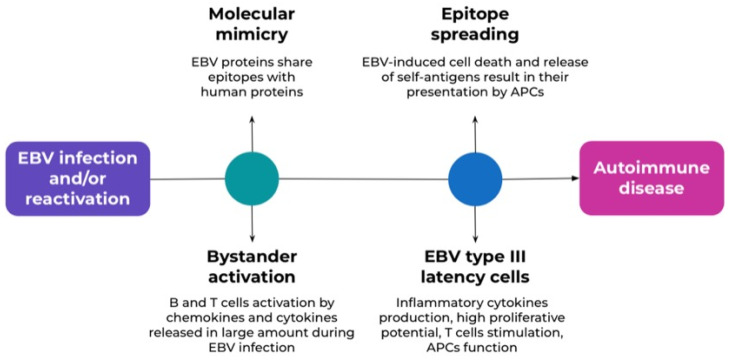
The main pathways of autoimmunity associated with EBV infection.

The development of autoimmune diseases is a complex process influenced by distinct mechanisms. It involves initiators, which trigger the onset of autoimmune responses, as well as predisposing factors that increase cellular susceptibility to autoimmunity. The promoters play a critical role in the progression of autoimmune conditions. EBV can act both as an initiator and a promoter (Figure 1) [28]. There are three main pathways for the initiation of autoimmunity in response to the viral infection: the molecular mimicry pathway, the bystander activation pathway, and the epitope spreading pathway. In the bystander pathway, B and T cells are activated in an antigen-independent manner by signals that promote an inflammatory environment. In the epitope spreading pathway, EBV induces cell death and the release of self-antigens. That results in their uptake and presentation by antigen-presenting cells (APCs). This process leads to de novo activation of autoreactive T cells that attack self-epitopes. The described mechanisms may occur because of EBV infection and its reactivation, causing autoimmunity [2,26].

Molecular mimicry is based on the structural similarity between a foreign antigen and self-antigens, which activate autoreactive T or B lymphocytes in a susceptible individual. It is presumed that EBV proteins share epitopes with human proteins, which causes an abnormal immune response. During the process of EBV replication, a substantial number of viral antigens are expressed, stimulating the production of autoantibodies. That leads to the accumulation of immune complexes and subsequent tissue damage. Viral nucleic acids and other pathogen/damage-associated molecular patterns can interact with pattern recognition receptors. That results in the release of proinflammatory cytokines and thereby activation of dendritic cells (DCs) and B lymphocytes or intracellular pathways in plasmacytoid DCs [22]. Munir et al. revealed that various EBV antigens, including EBNA-1 and BOLF1, share high sequence homology with human proteins. In RA, anti-CCP antibodies have been shown to cross-react with citrullinated EBV peptides, contributing to synovial inflammation. Similarly, in SLE, antibodies directed against EBNA-1 can recognize nuclear autoantigens such as Sm B/B′ and double-stranded DNA. In MS, EBNA-1–specific antibodies and T cells demonstrate cross-reactivity with myelin components like GlialCAM and PLP, supporting EBV’s involvement in CNS demyelination [30].

In both primary and chronic EBV infections, type III latency cells are present and can induce autoimmune reactions. These cells produce inflammatory cytokines, exhibit high proliferative potential, promote potent T-cell stimulation, and function as APCs and facilitate viral and host antigen presentation. The presentation of viruses or autoantigens by B cells during EBV type III latency stimulates the proliferation of autoreactive B cells and T cells, which may be enhanced by cellular cytokine production. The EBV gene product EBNA2, which is a characteristic of type III latency cells, is associated with risk loci for SLE, MS, RA, Crohn’s disease (CD), type 1 diabetes (T1D), juvenile idiopathic arthritis, and celiac disease [19,28,31]. EBV type III latency cells exhibit antiviral activity. They can induce the production of type I IFN, a significant factor in the pathogenesis of SLE. In addition, there is much evidence in the literature confirming the involvement of the EBV virus in the pathogenesis of MS. A major risk factor for MS maps to the class II region of the human leukocyte antigen (HLA) gene cluster, with the strongest contribution from HLA-DRB1*15:01. HLA-DRB1*15:01 is a co-receptor for EBV and has been associated with impaired immune surveillance of EBV in a humanized mouse model. MS patients exhibit reduced T-cell responses to type III latency cells. Type III latency cells can exacerbate experimental autoimmune encephalomyelitis in xenografted mice. Some MS patients have demonstrated benefits from autologous therapy with EBV-specific T cells, especially against EBV latency antigens, including LMP1 [28].

EBV has an important role in chronic immunological activation. It contributes to the development of autoimmune reactions. EBV is linked to higher antibody levels, increased viral load, and decreased T-cell responses in SLE, RA, and MS. In MS, EBV-infected B cells aggregate in the brain and produce ectopic lymphoid follicles, indicating ongoing immunological activity. In addition, EBV may increase the production of proinflammatory cytokines and allow autoreactive T-cell infiltration into the CNS. These findings indicate that chronic EBV infection may disrupt the immune response, particularly in genetically predisposed individuals, ultimately leading to the etiology of autoimmune disorders [11].

### 5.7. T-Bet+ B Cells

B cells expressing the transcription factor T-bet (T-bet+ B cells) appear to play an important role in autoimmunity. That has been particularly demonstrated in SLE and MS. T-bet is upregulated in B cells after interaction with IFN-γ-producing follicular helper T cells in the embryonic centers in association with B-cell receptor, Toll-such as receptor (TLR) 7, and/or TLR9 signaling. Patients with SLE, MS, and RA have higher percentages of T-bet+ B cells in their circulation. Several studies have demonstrated that T-bet+ B cells are a major source of autoantibodies in SLE. T-bet+ B cells are potent APCs, which are deemed crucial for directing CD4+ T cells to the brain in MS. These T cells may contribute to the disruption of the blood-brain barrier by allowing other cells, such as CXCR3+ B cells, to enter the tissue. It appears that T-bet+ B cells may also play a role in RA, as levels of T-bet+ B cells in peripheral blood have been associated with disease activity and have therefore been suggested as a potential marker of treatment response in RA patients. In CD, T-bet+ B cells have been associated with the active inflammatory state, exhibiting elevated levels of proinflammatory cytokines such as IFN-γ and generating IgG antibodies. A population of activated B cells expressing T-bet and other T-bet-related markers has been identified in the blood of individuals with T1D. The precise function and induction of T-bet+ B cells in these types of autoimmune diseases remain unclear [31].

### 5.8. Coinfection with Other Viruses

Emerging evidence indicates that EBV-seropositive individuals co-infected with other viruses, particularly CMV, may experience compounded immune dysregulation. Coinfection can exacerbate molecular mimicry events, as viral proteins from both EBV and CMV share linear amino acid motifs with host proteins, increasing the risk of cross-reactive antibody and T-cell responses [32,33]. Simultaneous activation of multiple latent viruses amplifies bystander activation of autoreactive lymphocytes via pro-inflammatory cytokine cascades and PRR signaling, lowering the activation threshold for self-reactive B and T cells [32]. Ebrahimi et al. highlight that EBV coinfections with CMV, HIV, or HPV can intensify dysregulated host immunity, thereby promoting autoimmunity and virus-associated malignancies. Indeed, such coinfection scenarios may potentiate epitope spreading and broaden the autoreactive repertoire beyond EBV monoinfection [34]. In children with multisystem inflammatory syndrome (MIS-C), SARS-CoV-2 infection leads to elevated TGF-β levels that impair T cell function, enabling EBV reactivation and highlighting a pathogenic role for SARS-CoV-2 and EBV coinfection in driving hyperinflammatory responses [35].

### 5.9. Genetic Predispositions

HLA class II alleles participate in modulating individual susceptibility to EBV-associated autoimmune diseases. Specific alleles such as HLA-DRB1*15:01, HLA-DQB1*06:02, and HLA-E*01:01 are connected with MS, SLE, RA, and SjS. In MS, individuals carrying the HLA-DRB1*15:01 allele show an increased humoral response to EBNA-1. This genetic background synergizes with a history of IM to significantly elevate disease risk [36,37]. In RA, the β-chain of HLA-DR4 exibits similarity to the gp110 amino acid sequence–QKRAA. This alters the reactivity of B and T cells toward self-proteins [38]. Susceptibility to SLE is associated with HLA-DRB1*15:01 and HLA-DR3, which facilitate the presentation of neoself-antigens following EBV reactivation, triggering autoreactive T cell responses [39]. These findings underscore the multifaceted role of HLA alleles in shaping the host immune response to EBV and in influencing the risk, clinical course, and immunopathology of the autoimmune diseases.

## 6. EBV and Autoimmune Diseases

### 6.1. Inflammatory Bowel Diseases

Inflammatory bowel diseases (IBDs) refer to both CD and ulcerative colitis (UC) [40]. Due to the use of immunomodulators, there has been growing evidence assessing the relationship between the occurrence of IBDs and EBV infection [5]. Nandy et al. demonstrated that individuals who later developed Crohn’s disease showed significantly higher seropositivity for EBV—particularly anti-EBNA1 antibodies—up to 7 years before diagnosis. Their findings suggest that prior EBV exposure and the strength of the immune response to EBNA1 may serve as early biomarkers and possible contributors to CD pathogenesis [41].

The EBV infection can lead to the development of superimposed viral colitis and progress to lymphoproliferative disorders. This may occur in the remission due to the removal of immunosuppressants. It is advised to screen patients for EBV infection prior to starting immunosuppressive treatment. During the therapy, the patient’s EBV status should be monitored. Individuals who had negative EBV test results are at risk of primary infection. The development of an IBD exacerbation and a worse prognosis are associated with EBV infection. Additionally, clinicians find it challenging to diagnose IBD and EBV-associated lymphoproliferative diseases due to overlapping symptoms and similar endoscopic findings [5].

The use of thiopurines increases susceptibility to viral infections [42]. Varicella-zoster virus, cytomegalovirus (CMV), and EBV infections can result in hemophagocytic lymphohistiocytosis. The risk of thiopurine-induced lymphomas (TIL) can be decreased by limiting the use of thiopurines in EBV-negative individuals. That refers particularly to young males and older men [42]. The incidence of TIL doubles in men treated with thiopurines and increases with age [42,43,44].

Beaugerie et al. gathered guidelines for the management of IBD in individuals who had lymphomas associated with EBV as their initial malignancy. Anti-TNF medications, methotrexate, tofacitinib, and thiopurines are not advised as first-choice medications. It appears that the use of ustekinumab and vedolizumab is tolerable. It remains unclear if anti-TNF drug monotherapy contributes to lymphoma formation [42].

Loosen et al. performed a cohort study involving 15,931 individuals suffering from IM and 15,931 healthy individuals. Their findings indicate that IM may cause an increase in the incidence of CD rather than UC. This dependence is particularly evident in women and young people (aged 14 to 20) (Table 2) [45].

### 6.2. Diabetes Mellitus Type I

Observations from the 1990s revealed that the HLA-DQ8 chain, which is associated with T1D, contains a specific five-amino acid sequence (GPPAA). This sequence was also found in the EBNA3C protein, encoded by the BERF4 gene of the EBV. Moreover, in the EBNA3C protein, the GPPAA sequence is present in six consecutive repeats, highlighting a potential structural or functional significance. In this study, the material was sera from healthy individuals, families with a history of T1D, children who had just been diagnosed with T1D, and individuals with acute EBV infection. In two out of seven individuals with acute EBV infection, antibodies against the peptide derived from the EBV virus were developed. These individuals were diagnosed with T1D. The result of the study is therefore a relationship between the EBV and T1D due to the molecular mimicry [46].

Although viral diseases are involved in the pathogenesis of diabetes [47], Wang and Liao did not show an increased risk of diabetes in patients infected with EBV [48]. In turn, Mohammed et al. revealed a significant difference in the presence of anti-EBV IgM antigen (*p* = 0.043) between patients with T1D and the control group. Moreover, almost 43% of patients with diabetes had anti-EBV IgG antigen, while none of the healthy individuals had this antigen detected. The viral genome was present in 15 of 56 people with diabetes, while in the control group the genome was not detected [49].

Chen et al. presented a case report of a 73-year-old patient who was hospitalized due to a drug-induced rash. During hospitalization, the patient developed fulminant T1D, which was attributed to a drug hypersensitivity reaction. Furthermore, the onset of diabetes was concurrent with an EBV infection, suggesting a potential interplay between the hypersensitivity reaction and viral infection in the disease progression [50]. Fulminant T1D is characterized by a rapid onset of ketoacidosis within a few days of the onset of hyperglycemia. The pathogenesis is often associated with viral infections, therefore, diagnostics for viral infections were performed. The EBV DNA titer was considered significant (4.744 × 10^4^ copies/mL [reference range, <1 × 10^3^ copies/mL]). The absence of infection with other viruses (parvovirus, CMV, rubella virus, adenovirus, respiratory syncytial virus, influenza virus, and parainfluenza virus) was confirmed. During the observation, it was determined that the coexistence of drug hypersensitivity and EBV infection played a key role in the induction of diabetes. While this particular case is rare, both drug hypersensitivity and EBV infection are relatively common in the general population, highlighting their potential contribution to disease development (Table 2) [50,51].

EBV-seropositive individuals show a stronger immunomodulatory response to teplizumab treatment in type 1 diabetes, characterized by increased regulatory T cells and reduced activation signaling in adaptive immune cells. EBV influences immune cell signaling pathways, potentially enhancing the therapeutic effect of teplizumab [35].

### 6.3. Systemic Autoimmune Diseases

Systemic autoimmune diseases (SADs) are characterized by the inflammation of the connective tissue. SADs encompass the relatively prevalent RA and more uncommon conditions such as SjS, SLE, and systemic scleroderma (SSc) [2]. It has been reported that individuals with SADs exhibit elevated levels of antibodies against EBV antigens [22]. Chronic or recurrent EBV infection of epithelial cells has been associated with SLE and SjS, whereas chronic or recurrent infection of B cells has been linked to RA. Since EBV can shuttle between epithelial and B cells, SADs often occur as overlapping syndromes [2]. Many risk loci in SLE and RA are occupied by EBNA2 [23,52]. Studies showed that EBV can cause SLE through molecular mimicry between EBNA1 and C1q, SmB, SmD, Ro, dsDNA, and epitope spreading. In RA, this mechanism involves molecular mimicry between EBNA1 and joint proteins, bystander activation, and chronic recurrent infection of joint epithelial cells and synovial B cells. SSC may result from aberrant activation of TLR-like antiviral responses. Meanwhile, SjS is associated with molecular mimicry between EBNA2 and Ro-60, as well as between EBER-1 and EBER-2 and La [26].

SLE is characterized by a range of symptoms, including leukopenia, thrombocytosis, skin rash, UV sensitivity, mucosal ulcerations, alopecia, pleuritis, nephritis, myositis, arthritis, vasculitis, and neuropsychiatric manifestations. The disease may have a relapsing or remitting course [2]. In the study by Laurynenka et al. all patients with SLE were infected with EBV. They tested positive for both VCA IgG and EBNA1 IgG antibodies, which were more common than in the control group. The risk of developing SLE associated with EBNA1 was estimated at 89.7%. For EBV infection overall, the risk reached 100%. These findings suggest that the immune response to EBNA1 may play a key role in the development of autoimmunity in SLE [53]. Sternbæk et al. examined sera from patients with SAD and healthy controls for the presence of IgM, IgA, and IgG antibodies binding to 11 EBV antigens (EBNA1, EBNA2, BALF5, EA, BALF2, EA/R, VCA p18, VCA p23, gB, gP350, and gP42). Results showed that patients with SAD had increased levels of antibodies. In SLE, elevated levels of IgA for EAD were found, whereas in RA, increased levels of IgM for several EBV antigens were detected [19]. In their cross-sectional study, França et al. examined 92 patients with SLE and 27 with RA. The active phase of infection was confirmed by the detection of EBV DNA in 40.29% of examined patients, of whom 45.65% suffered from SLE and 25.92% from RA [24]. Guta et al. examined 115 patients with SLE to determine EBV and CMV infection. The vast majority of SLE patients were infected with EBV (98.26%). Active EBV infection was detected in 15.65% of SLE patients and chronic, persistent infection in 53.91%. The most common serological profile in SLE patients was EBNA1 IgG, (+) EAD IgG, (+) and VCA IgM (−) [54]. Banko et al. showed that the presence of EAD IgG antibodies was associated with a 24-fold higher probability of developing SLE. Higher titers of EAD IgG antibodies were identified as an independent factor associated with lymphopenia, while higher titers of VCA IgG antibodies and positive rheumatoid factor (RF) with alopecia in SLE [55]. Jog et al. found that higher levels of VCA IgG and EAD IgG antibodies were associated with a higher risk of developing SLE in at-risk individuals [56]. Aygun et al. examined 70 patients with juvenile SLE (jSLE), 14 patients with juvenile SSc (jSSc), and 44 healthy controls. EBV VCA was detected in 84.2% of jSLE patients, 85.7% of jSSc patients, and 36.3% of healthy individuals. IgG EAD levels were significantly higher in jSLE patients compared to the other groups. EBV VCA positivity was associated with malar rash and immunological abnormalities [57]. According to the study performed by Izadi et al. SLE patients had significantly higher EBV load and transcript levels of TLR7, IFN-α, and TLR9 compared to controls. Furthermore, plasma IFN-α levels and EBNA1 IgG antibodies were significantly higher in this group of patients [58]. In another study, Afrasiabi et al. identified 79 SLE risk locus-gene pairs putatively interacting with EBV infection. Ten risk genes were targeted by EBNA2, and most of them were also associated with EBV DNA copy number and EBV gene expression level [52]. Another study by Chen et al. revealed that SLE patients with lytic EBV infection had higher disease activity and took longer to achieve remission [59]. In the study by Ming et al. SLE patients who responded well to treatment had a transformation of EBV DNA from positive to negative, so EBV DNA seroconversion may be an indicator reflecting the response to the applied therapy [60]. According to Truszewska et al. SLE patients with chronic kidney disease (CKD) had higher EBV loads compared to patients without CKD [61].

SjS is characterized by B-cell hyperactivation and inflammation in salivary and lacrimal glands. B cells produce autoantibodies against SjS-associated proteins SSA and SSB, which causes damage to the epithelium of the exocrine glands. That leads to xerophthalmia and xerostomia [62]. Barcelos et al. showed that EBV infection contributes to the activation of lymphocytes in ectopic germinal centers, causing autoimmune epithelial inflammation and glandular destruction in SjS. In this study, patients with SjS had a higher prevalence of IgG EAD antibodies [63]. Similarly, in a case-control study by Xuan et al. patients with SjS had a significantly higher frequency of positive IgG EAD antibodies and their high titers. The level of IgG VCA antibodies was significantly higher in patients with SjS compared to the control group. In addition, IgG EAD antibodies were associated with low levels of C3 and C4 in patients with SjS [64].

Clinical manifestations of RA include joint swelling and pain due to inflammation of the synovium, which leads to excessive deposition of connective tissue and bone erosion. Most patients with RA show the presence of RF, anti-CCP antibodies, and anti-nuclear antibodies (ANAs) [2]. The study by Sorgato et al. aimed to assess the relationship between EBV and the presence of RA and its connection with SjS. EBV infection was more common in patients with RA and RA/SjS than in the control group. Patients with RA and RA/SjS had a higher number of EBV DNA copies. Moreover, EBV DNA was associated with the results of the Schirmer test [65]. Munir et al. studied 85 women and 15 men with RA. EBV was detected in 45% of patients with RA. Of the 100 examined patients, 43% were seropositive for RA. There was a significant correlation with a family history of RA in EBV-positive individuals [66]. Li et al. assessed the levels of IgG, IgA, and IgM against EBV gps (gP350, gP42, gHgL, and gB) in serum samples collected from RA and SLE patients and found that RA and SLE patients showed statistically significant increases in the levels of 8 and 11 glycoprotein antibodies, compared to the control group. The significant diagnostic markers of RA were gP350 IgG, gP350 IgA, gH/gL IgM, and gP42 IgA, while the markers of SLE included gP350 IgG, gP350 IgA, gH/gL IgA, and gP42 IgM. Increased levels of gPs antibodies suggest that EBV reactivation and replication occurred in patients with SAD, which may be a promising diagnostic biomarker (Table 2) [22].

### 6.4. Autoimmune Thyroid Diseases

Studies indicate that the mechanism by which EBV contributes to the onset of autoimmune thyroid diseases (AITD) such as Graves’ disease (GD) and Hashimoto’s thyroiditis (HT) is linked to latent EBV type III infection of thyroid epithelial cells and infiltrating lymphocytes. This may lead to the cross-reactivity of antibodies against EBER and LMP1 with host proteins through molecular mimicry [26]. De Almeida et al. found that 2 of 17 thyroid tissues (11.8%) from patients with AITD (2 with GD and 15 with HT) were EBV-positive and showed high EBER expression in lymphoid tissue [67]. Hamad et al. demonstrated that LMP1 was detected in 11.1% of patients with HT in Sudan [68]. The study by Pyzik et al. included 39 untreated patients with newly diagnosed GD. The findings revealed a significantly higher prevalence of EBV copies in peripheral blood mononuclear cells (PBMCs) in patients with GD compared to the control group [69]. Kirino et al. described a case report of an 8-year-old girl diagnosed with T1D and GD simultaneously during primary EBV infection [70]. Tamoto et al. analyzed the presence of thyrotropin (TSH) receptor antibodies (TRAb)(+) cells, EBV(+) cells, and TRAb(+) EBV(+) cells in PBMCs from 29 healthy or subclinical children without GD and one cord blood sample. The findings revealed that low levels of TRAb production were observed in EBV primary infection and lytic reactivation in children who exhibited no symptoms of IM. Moreover, the populations of TRAb(+) and EBV(+) cells were modest during primary infection but had the potential to increase upon repeated EBV lytic reactivation. This may provide an explanation why GD typically manifests in young adults, not in infants [71]. The study conducted by Nagata et al. showed that EBV reactivation resulted in the generation of IgM-dominant TRAb antibodies. These antibodies did not inhibit TSH binding to TSH receptors and transmit hormone-producing signals; however, they destroyed thyroid follicular epithelial cells via complement, resulting in the development and exacerbation of GD (Table 2) [72].

**Table 2:** The summary of the cited reports on EBV in the pathogenesis of autoimmune diseases.

Study	AD	Results
Loosen et al. Infectious mononucleosis is associated with an increased incidence of Crohn’s disease: results from a cohort study of 31 862 outpatients in Germany	IBD	- IM causes an increase in the incidence of CD rather than UC. This dependence is particularly evident in women and young people (aged 14 to 20).
Zhang et al. Impact of Epstein-Barr virus infection in patients with inflammatory bowel disease	IBD	- EBV infection is prevalent among individuals with IBD and has the potential to cause numerous complications, such as EBV-related superimposed colitis and lymphoproliferative disorders.
Mohammed et al. The possible Association between Epstein-Barr Virus and Type 1 Diabetes Mellitus	T1D	- There was a significant difference between patients with T1D and healthy controls in the presence of anti-EBV IgM. - Nearly 43% of patients with T1D had anti-EBV IgG, unlike healthy individuals who did not. - The viral genome was present in 15 of 56 patients with T1D, while in the control group the genome was not detected.
Chen et al. Fulminant Type 1 Diabetes Mellitus Associated With Drug Hypersensitivity and Epstein-Barr Virus Infection: A Case Report	T1D	- Case report of a 73-year-old patient hospitalized for a drug-induced rash. She developed fulminant T1D, caused by a drug hypersensitivity reaction and concomitant EBV infection.
Laurynenka et al. A High Prevalence of Anti-EBNA1 Heteroantibodies in Systemic Lupus Erythematosus (SLE) Supports Anti-EBNA1 as an Origin for SLE Autoantibodies	SLE	- All examined patients with SLE were infected with EBV. - Patients with SLE had higher titers of VCA IgG and EBNA1 IgG compared to the control group. - EBNA1 IgG was present in >99% of SLE patients.
Banko et al. Epstein-Barr virus infection as potential indicator of the occurrence and clinical presentation of systemic lupus erythematosus	SLE	- EAD IgG was associated with a 24-fold higher probability of developing SLE. - Higher titers of EAD IgG were identified as an independent factor associated with lymphopenia, while higher titers of VCA IgG and positive RF with alopecia in SLE.
Barcelos et al. Association between EBV serological patterns and lymphocytic profile of SjS patients support a virally triggered autoimmune epithelitis	SjS	- EBV infection contributes to the activation of lymphocytes in ectopic germinal centers, causing autoimmune epithelial inflammation and glandular destruction in SjS. - Patients with SjS had a higher prevalence of EAD IgG.
Xuan et al. Serological Evidence for the Association Between Epstein-Barr Virus Infection and Sjögren’s Syndrome	SjS	- Patients with SjS had significantly higher titers of EAD IgG and VCA IgG compared to the control group. - The presence of EAD IgG was associated with low levels of C3 and C4 in patients with SjS.
Li et al. Evaluation of serum Epstein-Barr virus envelope glycoproteins antibodies and their association with systemic autoimmune diseases	RA	- RA and SLE patients showed statistically significant increased levels of EBV gp antibodies. - The significant diagnostic markers of RA were gP350 IgG, gP350 IgA, gH/gL IgM, and gP42 IgA.
Munir et al. Frequency and association of Epstein-Barr Virus genotype in rheumatoid arthritis patients of Khyber Pakhtunkhwa, Pakistan	RA	- EBV was detected in 45% of patients with RA. - There was a significant correlation with a family history of RA in EBV-positive individuals.
Pyzik et al. Does the Epstein-Barr Virus Play a Role in the Pathogenesis of Graves’ Disease?	AITD	- Untreated patients with newly diagnosed GD had a significantly higher prevalence of EBV copies in PBMCs compared to the control group.
Nagata et al. Epstein-Barr virus reactivation in peripheral B lymphocytes induces IgM-type thyrotropin receptor autoantibody production in patients with Graves’ disease	AITD	- EBV reactivation resulted in the generation of IgM-dominant TRAb antibodies. - TRAb-IgM antibodies did not inhibit TSH binding to TSH receptors and did not transmit hormone-producing signals; however, they destroyed thyroid follicular epithelial cells via complement, resulting in the development and exacerbation of GD.
Cavalcante et al. Epstein-Barr virus persistence and reactivation in myasthenia gravis thymus [62]	MG	- All 17 patients with MG had an active EBV infection, while none of the 6 people from the control group was infected. - In 12 patients’ thymuses, cells expressing EBER were detected, and in 16, EBV latency proteins (EBNA2, LMP1, and LMP2A)were detected.
Cavalcante et al. Toll-like receptors 7 and 9 in myasthenia gravis thymus: amplifiers of autoimmunity? [64]	MG	- In the thymus of MG patients, significant dysregulation of TLR7 and TLR9 was observed. It correlated with the presence of EBV. - TLR7 and TLR9 may increase the risk of aberrant activation and survival of pathological B lymphocytes, leading to an autoimmune reaction.
Zachova et al. Role of Epstein-Barr Virus in Pathogenesis and Racial Distribution of IgA Nephropathy [65]	IgAN	- Patients with IgAN had a higher number of lymphoblasts/plasmablasts that were surface-positive for IgA, infected with EBV, and exhibited heightened expression of homing receptors.
Sato et al. Acute kidney injury in an adult patient with IgA nephropathy and chronic replicative Epstein-Barr virus infection [67]	IgAN	- Case report of an 80-year-old woman with IgA nephropathy and chronic replicative EBV infection who developed acute kidney injury.
Lanz et al. Clonally expanded B cells in multiple sclerosis bind EBV EBNA1 and GlialCAM [13]	MS	- A Monoclonal antibody that binds to the EBNA1 AA386–405 epitope and exhibits cross-reactivity with GlialCAM was found in the cerebrospinal fluid of an MS patient. - Immunization of mice with the EBNA1 epitope resulted in increased demyelination. - 20% to 25% of MS patients have antibodies against GlialCAM and EBV EBNA1.
Al-Obaidi et al. The potential role of Epstein Barr virus in multiple sclerosis molecular and serological study [71].	MS	- EBNA1 IgG antibodies were positive in 51.7% of MS patients and 39.2% of control subjects. - The median level of EBNA1 IgG antibodies in MS patients and control subjects was 81.08 U/mL and 67.73 U/mL, respectively. - EBNA1 IgG levels were significantly higher in younger age groups. - There were no significant differences in EBNA1 IgG levels in patients treated with first- and second-line therapy, while the median level in untreated (newly diagnosed) patients was higher.
Jacobs et al. Systematic review and meta-analysis of the association between Epstein–Barr virus, multiple sclerosis and other risk factors [68]	MS	- Individuals with high titers of anti-EBNA antibodies and the HLA-DRB11501 allele have a significantly increased risk of developing MS. - 92.1% of MS patients were EBV seropositive, compared to 81.4% of controls. - The OR for MS in EBV-seropositive patients was 3.92, highlighting the strong association between EBV infection and MS.
Bjornevik et al. Longitudinal analysis reveals high prevalence of Epstein-Barr virus associated with multiple sclerosis [4]	MS	- EBV infection increases the risk of MS 32 times (HR = 32.4; 95% CI: 4.3–245.3; *p* < 0.001). - EBV infection contributes to the increase in light neurofilament levels even before the onset of clinical symptoms.

AD—autoimmune disease; EBV—Epstein-Barr virus; IBD—inflammatory bowel disease; IM—infectious mononucleosis; CD—Crohn’s Disease; UC—Ulcerative Colitis; T1D—Type 1 Diabetes; Ig—immunoglobulin; SLE—systemic lupus erythematosus; VCA—viral capsid antigen; EAD—Early Antigen Diffuse, RF—rheumatoid factor, SjS—Sjögren’s syndrome, RA—rheumatoid arthritis, gP—glycoprotein; AITD—autoimmune thyroid disease; GD—Graves’ Disease; PBMCs—Peripheral Blood Mononuclear Cells; TRAb—Thyrotropin Receptor Antibodies; TSH—Thyrotropin; MG—Myasthenia Gravis; EBNA2—Epstein-Barr virus Nuclear Antigen 2; LMP1—Latent Membrane Protein 1; LMP2A—Latent Membrane Protein 2A; TLR7—Toll-like Receptor 7; TLR9—Toll-like Receptor 9; IgAN—IgA Nephropathy; MS—Multiple Sclerosis; EBNA1—Epstein-Barr virus Nuclear Antigen 1; HLA—Human Leukocyte Antigen; OR—Odds Ratio.

### 6.5. Myasthenia Gravis

Myasthenia gravis (MG) is a disease of the nervous system with the presence of autoantibodies against components of the neuromuscular junction. The antibodies target the acetylcholine receptor (AChR) or the tyrosine kinase (MuSK). MG is associated with thymus pathology. The inflammation, excessive expression of TLRs, and formation of germinal centers (GC) are present in the myasthenic thymus. This clinical presentation may indicate viral etiology. Excessive expression of TLRs leads to autoimmunization of AChR [73,74]. However, the exact pathophysiology of immunization against self-antigens is still unclear.

Cavalcante et al. examined thymus tissue from thymectomized patients with MG and a control group of cardiopathic patients, with tissue collected during heart surgery. Their findings revealed that all 17 patients in the MG group had an active EBV infection. In contrast, none of the 6 individuals in the control group tested positive for EBV infection. In 12 patients’ thymuses, cells expressing EBER were detected, and in 16, EBV latency proteins (EBNA2, LMP1, and LMP2A) [74].

In another study, researchers attempted to detect the EBV genome using the Southern blot method on six thymuses from patients with myasthenia gravis and thymoma, one patient with thymoma without myasthenia gravis, and three healthy control thymuses. The results were as follows: 2 of 2 with MG, 1 of 1 without MG, 2 of 4 thymic lymphoid hyperplasia (TLH) with MG, and 1 of 1 TLH without MG were positive for the EBV genome. It should be emphasized that this was one of the first studies dealing with the relationship between EBV and myasthenia. The techniques and methods of tissue storage used in the study are no longer optimal [75].

Taking the TLRs under the microscope, it is worth citing another study by Cavalcante et al. They emphasized significant dysregulation of TLR7 and TLR9 in the thymuses of MG patients, which correlated with the presence of EBV. These receptors may increase the risk of abnormal activation and survival of pathological B lymphocytes, which leads to an autoimmune reaction. This happens because activation of the receptors leads to the production of type I IFN and proinflammatory cytokines. A closer look at this issue may contribute to the development of the treatment model targeting this mechanism (Table 2) [76].

### 6.6. IgA Nephropathy

IgA nephropathy (IgAN) is the most prevalent type of primary glomerulonephritis worldwide [77]. The pathogenesis of IgAN is associated with poorly galactosylated IgA1, an antigen recognized by naturally occurring anti-glycan antibodies. As a result, the nephritogenic circulating immune complexes are produced [78]. Human B cells that have been infected in vitro with EBV secrete galactose-deficient IgA1. Zachova et al. examined peripheral blood B cells from adult patients with IgAN. The examined group exhibited increased lymphoblasts and plasmablasts that were surface-positive for IgA, infected with EBV, and demonstrated heightened expression of homing receptors. Upon polyclonal stimulation, these cells produced more galactose-deficient IgA1 compared to the cells from healthy controls. In healthy African Americans, EBV was mainly detected on IgM- and IgD-positive cells. It is notable that the majority of African Americans contract EBV within two years from their birth. During that period, the IgA system is naturally deficient, as evidenced by low serum IgA levels and a limited number of IgA-producing cells. EBV infects cells that produce immunoglobulins other than IgA. It was found that EBV-infected IgA+ cells were a source of galactose-deficient IgA1 and a basis for the expression of relevant homing receptors. The effect of racial-specific disparities in EBV infection on the naturally delayed maturation of the IgA system explicates the racial disparity in the prevalence of IgAN [77]. Sato et al. described a case of an 80-year-old woman with IgA nephropathy and chronic replicative EBV infection who developed acute kidney injury (Table 2) [79].

### 6.7. Multiple Sclerosis

MS is a chronic autoimmune disease that affects the central nervous system. It is characterized by inflammation, demyelination, and neurodegeneration. The exact cause of MS is still unknown, but there is growing evidence suggesting a potential role of the EBV in the development and progression of the disease [80]. Lanz et al. demonstrated molecular mimicry between EBV EBNA1 and the glial cellular adhesion molecule GlialCAM. They have identified a monoclonal antibody isolated from the cerebrospinal fluid of a patient with MS that binds to the EBNA1 AA386–405 epitope and exhibits cross-reactivity with GlialCAM. Furthermore, immunization of mice with the EBNA1 epitope resulted in increased demyelination [15].

The interaction between EBNA titers and the HLA-DRB11501 genotype has been studied in the context of MS. Individuals with high anti-EBNA antibody titers and the HLA-DRB11501 allele have a significantly increased risk of developing MS. Specifically, the odds ratio (OR) for MS in HLA-DRB11501-positive individuals with high EBNA titers is 7.90 compared to 3.04 in those without the allele. These findings indicate a synergistic effect between EBNA titers and the HLA-DRB11501 genotype, suggesting that genetic predisposition and viral infection jointly contribute to MS risk [80].

IM, caused by EBV, is another factor interacting with the HLA-DRB11501 genotype to influence MS susceptibility. A history of IM is associated with a higher risk of MS, particularly in individuals with the HLA-DRB11501 allele. The OR for MS in HLA-DRB1*1501-positive individuals with a history of IM is 5.11, compared to 1.22 in those without the allele [80].

Smoking is a well-known risk factor for MS, and its interaction with EBV infection amplifies this risk. The combined presence of high EBNA titers and smoking significantly increases the likelihood of developing MS. The OR for MS in smokers with high EBNA titers is 2.76 compared to 1.16 in non-smokers with low EBNA titers. The additive interaction analysis showed a substantial attributable proportion, indicating that smoking and EBV infection together create a higher risk environment for MS development [80,81].

Vitamin D deficiency has been implicated in MS risk. However, studies have not consistently demonstrated a significant interaction between vitamin D levels and anti-EBNA titers. The limited data suggest no clear synergistic effect between low vitamin D levels and EBV infection in increasing MS risk [80].

EBV seropositivity is significantly higher in individuals with MS compared to healthy controls. A meta-analysis including 56 studies found that 92.1% of people with MS were EBV-seropositive, compared to 81.4% of control subjects. The OR for MS in EBV seropositive individuals was 3.92, underscoring the strong association between EBV infection and MS. This consistent finding across multiple studies reinforces the hypothesis that EBV plays a crucial role in MS pathogenesis, potentially triggering the disease in genetically predisposed individuals. Furthermore, studies have shown that the risk of MS increases with the age of EBV infection, suggesting that later infections may be more likely to trigger the disease [80,82]. In the case-control study conducted by Al-Obaidi et al. EBNA1 IgG antibodies were positive in 51.7% of MS patients and 39.2% of control subjects. The median level of EBNA1 IgG antibodies in MS patients and control subjects was 81.08 U/mL and 67.73 U/mL, respectively. EBNA1 IgG levels were significantly higher in younger age groups. There were no significant differences in EBNA1 IgG levels in patients treated with first- and second-line therapy, while the median level in untreated, newly diagnosed patients was higher [83].

Bjornevik et al. conducted a landmark cohort study involving more than 10 million US soldiers. The research revealed that EBV infection is a major risk factor for MS. EBV infection increased the risk of MS 32 times (HR = 32.4; 95% CI: 4.3–245.3; *p* < 0.001). Other viruses, including cytomegalovirus, had no similar effect. In those who seroconverted to EBV, there was an increase in light neurofilament levels even before the onset of clinical symptoms (*p* < 0.05). EBV infection preceded both the onset of symptoms and the first detectable pathological changes, suggesting that EBV is a major cause of MS (Table 2) [84].

### 6.8. Primary Immune Regulatory Disorders

EBV infections are an important clinical aspect in patients with primary immune regulation disorders (PIRD). Patients with a subgroup of disorders predisposing to hemophagocytic lymphohistiocytosis (HLH) are particularly at risk. PIRDs associated with EBV susceptibility result from defects in lymphocyte cytotoxicity. This leads to uncontrolled activation of macrophages and cytotoxic lymphocytes and excessive cytokine secretion. Diseases in this group include familial HLH, Chediak-Higashi syndrome, Griscelli syndrome type 2, and X-linked lymphoproliferative disorders such as X-linked inhibitor of apoptosis protein (XIAP) and SH2D1A deficiency. Patients with these disorders often present with fever, hepatosplenomegaly, cytopenia, and persistent or recurrent EBV infections. Clinical suspicion of PIRD, especially in children with severe, uncontrolled EBV infections or HLH, should prompt comprehensive immunological and genetic disorders [85].

## 7. Vaccination

Currently, no vaccine is approved for EBV. There are clinical trials in phase 1 or 2 underway on the gp350-Ferritin Nanoparticle Vaccine, the gH/Gl/gp42-Ferritin Nanoparticle Vaccine, mRNA-1189, mRNA-1195, OSU-2131, etc. [86] EBV vaccines hold promise for preventing primary EBV infection. They may also protect against diseases directly caused by the virus, such as infectious mononucleosis and post-transplant lymphoproliferative disorder. Additionally, they could help reduce the risk of conditions in which EBV acts as a cofactor. These include multiple sclerosis, certain EBV-positive lymphomas, and the above mentioned autoimmune diseases. Prophylactic strategies primarily aim to elicit strong neutralizing antibody responses that block viral entry into B cells, while therapeutic approaches focus on generating EBV-specific T cells capable of targeting infected or malignant cells. By interrupting the viral lifecycle or reactivation within B cells, vaccines may help prevent the downstream immunological events that lead to autoantibody production [87].

## 8. Conclusions

The EBV is equipped with many tools that allow it to infect efficiently, evade the host’s immune response, and maintain an excellent state of latency. Moreover, its ability to induce autoimmune processes in various ways, which we discussed above, leads to the pathogenesis of a wide range of diseases. This draws attention to the scale of the problem and calls for in-depth research into both disease prevention and individualized treatment in specific disease entities. Given that EBV spreads worldwide and contributes to both autoimmune diseases and several malignancies, the development of effective vaccines remains a high priority. Promising prophylactic strategies include multivalent mRNA-based vaccines and nanoparticle platforms that aim to elicit both neutralizing antibodies and EBV-specific T-cell responses to block primary infection and latency-associated complications. Precision strategies targeting viral mimicry and autoreactive immune pathways may help prevent epitope spreading and restore immune tolerance. As vaccine platforms evolve, global surveillance and equitable access will be essential to reduce the clinical burden of EBV-related diseases.

## Figures and Tables

**Table 1:** Serological parameters and their interpretation in the assessment of EBV infection status.

	Antibodies				
	Heterophile	VCA IgM	VCA IgG	EA-D	EBNA-1
Acute primary infection	+	+	+	+	−
Past infection	−	−	+	−	+
Past active infection	−	−	+++	+	+
EBV reactivation, Burkitt’s lymphoma, nasopharyngeal carcinoma	−	+/−	+	+/−	+
Seronegative	−	−	−	−	−

+—positive, −—negative, EA-D—Epstein-Barr virus early antigen diffuse component, EBNA-1—Epstein-Barr virus nuclear antigen 1, EBV—Epstein-Barr virus, VCA—viral capsid antigen.
